# Altered monocyte and fibrocyte phenotype and function in scleroderma interstitial lung disease: reversal by caveolin-1 scaffolding domain peptide

**DOI:** 10.1186/1755-1536-4-15

**Published:** 2011-07-01

**Authors:** Elena Tourkina, Michael Bonner, James Oates, Ann Hofbauer, Mathieu Richard, Sergei Znoyko, Richard P Visconti, Jing Zhang, Corey M Hatfield, Richard M Silver, Stanley Hoffman

**Affiliations:** 1 Division of Rheumatology and Immunology, Department of Medicine, Medical University of South Carolina, 171 Ashley Avenue, Charleston, SC 29425, USA; 2 Department of Regenerative Medicine and Cell Biology, Medical University of South Carolina, 171 Ashley Avenue, Charleston, SC 29425, USA

## Abstract

Interstitial lung disease (ILD) is a major cause of morbidity and mortality in scleroderma (systemic sclerosis, or SSc). Fibrocytes are a monocyte-derived cell population implicated in the pathogenesis of fibrosing disorders. Given the recently recognized importance of caveolin-1 in regulating function and signaling in SSc monocytes, in the present study we examined the role of caveolin-1 in the migration and/or trafficking and phenotype of monocytes and fibrocytes in fibrotic lung disease in human patients and an animal model. These studies fill a gap in our understanding of how monocytes and fibrocytes contribute to SSc-ILD pathology. We found that C-X-C chemokine receptor type 4-positive (CXCR4^+^)/collagen I-positive (ColI^+^), CD34^+^/ColI^+ ^and CD45^+^/ColI^+ ^cells are present in SSc-ILD lungs, but not in control lungs, with CXCR4^+ ^cells being most prevalent. Expression of CXCR4 and its ligand, stromal cell-derived factor 1 (CXCL12), are also highly upregulated in SSc-ILD lung tissue. SSc monocytes, which lack caveolin-1 and therefore overexpress CXCR4, exhibit almost sevenfold increased migration toward CXCL12 compared to control monocytes. Restoration of caveolin-1 function by administering the caveolin scaffolding domain (CSD) peptide reverses this hypermigration. Similarly, transforming growth factor β-treated normal monocytes lose caveolin-1, overexpress CXCR4 and exhibit 15-fold increased monocyte migration that is CSD peptide-sensitive. SSc monocytes exhibit a different phenotype than normal monocytes, expressing high levels of ColI, CD14 and CD34. Because ColI^+^/CD14^+ ^cells are prevalent in SSc blood, we looked for such cells in lung tissue and confirmed their presence in SSc-ILD lungs but not in normal lungs. Finally, in the bleomycin model of lung fibrosis, we show that CSD peptide diminishes fibrocyte accumulation in the lungs. Our results suggest that low caveolin-1 in SSc monocytes contributes to ILD via effects on cell migration and phenotype and that the hyperaccumulation of fibrocytes in SSc-ILD may result from the altered phenotype and migratory activity of their monocyte precursors.

## Background

Scleroderma (systemic sclerosis, SSc) is a complex autoimmune connective tissue disease involving inflammation and fibrosis of the skin, lungs and other internal organs. The main cause of morbidity and mortality in SSc is interstitial lung disease (ILD). Until recently, lung fibrosis was generally believed to result from the proliferation and activation of resident connective tissue fibroblasts [1]. However, recent studies have also indicated that fibroblasts can be derived from hematopoietic cells and by epithelial- or endothelial-mesenchymal transformation. In fact, the notion that matrix-producing cells could be derived from peripheral blood mononuclear cells (PBMCs) peripheral blood cells is not new. It was suggested by Metchnikov and others 100 years ago [2-5].

PBMCs play important roles in inflammation, fibrosis and wound healing because of their immune functions and because they are the progenitors of collagen-producing cells. The CD14^+ ^monocyte fraction contains precursors not only for macrophages but also for fibrocytes. Circulating connective tissue cell progenitors (fibrocytes) were described previously [6] as a subpopulation of PBMCs that express collagen together with hematopoietic cell surface markers (for example C11b, CD34 and/or CD45), but that do not express CD14. In addition, a population of CD45^+^/CD14^+^/collagen I-positive (ColI^+^) cells described as "collagen-producing monocytes" was recently observed at much higher levels in the peripheral blood of SSc patients than in control subjects [7].

Both monocytes and fibrocytes express on their surface the C-X-C chemokine receptor type 4 (CXCR4). CXCR4 mediates the migration of these cells in response to stromal cell-derived factor 1 (SDF-1, or CXCL12), which is expressed at high levels in injured human and mouse lung tissues [8]. In addition, fibrocytes contribute to tissue remodeling by producing high levels of cytokines, fibrogenic growth factors, extracellular matrix proteins and matrix metalloproteinase [1,8-12].

Caveolin-1 plays a central role in several signaling cascades in which it serves as a scaffolding protein that binds to a variety of kinases and thereby regulates their activity. As we have shown recently, caveolin-1 plays a crucial role in regulating monocyte signaling and function in SSc. We found PBMCs from SSc-ILD patients to be deficient in caveolin-1 and to overexpress CXCR4. The phenotype of low caveolin-1 and high CXCR4 expression can be mimicked in normal monocytes by transforming growth factor β (TGFβ) treatment [13]. Our data, together with data from other groups, strongly suggest that caveolin-1 is a key signaling molecule in the monocyte-fibrocyte-fibroblast lineage and is responsible for functional differences observed among cells isolated from SSc-ILD and idiopathic pulmonary fibrosis (IPF) patients compared to control subjects [14-16].

In the current study, we have extended our analysis of the roles of caveolin-1 and CXCR4 in regulating the functions of monocytes and fibrocytes and in the pathology of SSc-ILD. We find that SSc monocytes differ functionally and phenotypically from normal monocytes. They are hypermigratory in response to CXCL12 because of their lack of caveolin-1 and their overexpression of CXCR4. Fibrocytes and "collagen-producing monocytes" were detected in the blood and lungs of SSc-ILD patients, but not in normal subjects. The ability of the caveolin scaffolding domain (CSD) peptide to regulate CXCR4 expression and monocyte and fibrocyte migration *in vivo *and thereby to inhibit the progression of lung injury and/or fibrosis was demonstrated in bleomycin-treated mice, suggesting that CSD peptide may be a useful therapeutic agent in SSc-ILD.

## Results

### Fibrocytes in SSc-ILD lung tissue

**Figure 1:**
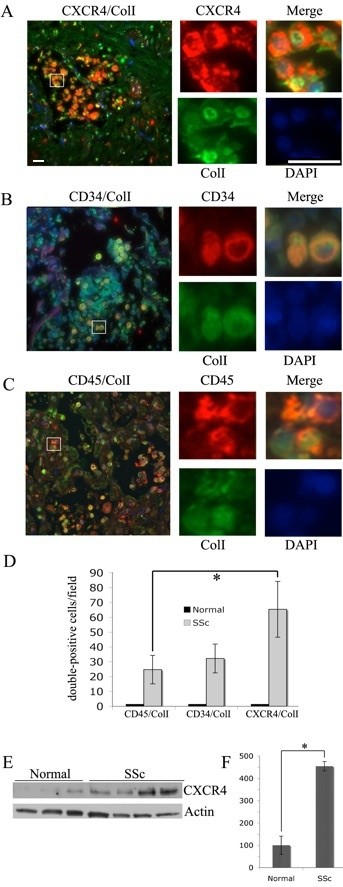
**Fibrocyte subsets in lung tissue of patients with systemic sclerosis-interstitial lung disease**. Fixed, paraffin-embedded lung tissue sections from normal adults and adults with systemic sclerosis-interstitial lung disease (SSc-ILD) were stained for CD34, CD45, C-X-C chemokine receptor type 4 (CXCR4) and collagen I (ColI). Nuclei were stained with 4',6-diamidino-2-phenylindole (DAPI) (blue). Left: low-magnification images. Right: high-magnification images. The portion of the low-magnification image that is presented at high magnification is boxed. **(A) **CXCR4 (red)/ColI (green). **(B) **CD45 (red)/ColI (green). **(C) **CD34 (red)/ColI (green). **(D) **The number of double-labeled cells per field was determined from images of sections obtained from seven SSc-ILD patients and four normal subjects, with six randomly chosen fields per sectio. *P < 0.05. CXCR4 expression was evaluated by performing Western blot analysis on extracts of three normal lung samples and four SSc-ILD lung samples **(E) **and then quantifying them densitometrically **(F) **using actin as a loading control. Bars = 10 μm.

Fibrocytes are cells expressing a hematopoietic marker (CD45, CD34) and a mesenchymal marker (ColI). The status of CD14 in these cells is controversial. In a recent report, the term "fibrocyte" was reserved for CD14^- ^cells, while CD14^+ ^cells were referred to as collagen-producing monocytes [7]. CXCR4 is also present on fibrocytes at high levels [12]. Fibrocytes and CXCR4^+^/ColI^+ ^cells are present in the lung tissue of IPF patients, but not in lung tissue from normal subjects [12]. Therefore, we looked for these cells in SSc-ILD lung tissue. We identified these cells in the lung tissue of all seven SSc-ILD patients examined (Figures 1A to 1C), but not in the lung tissue of healthy individuals. Fibrocytes and CXCR4^+^/ColI^+ ^cells were found both in established lesions (for example, Figures 1B and 1C) and at sites where active remodeling of alveoli was still occurring (for example, Figure 1A). When we compared the number of cells identified using different markers, we found considerably more CXCR4^+^/ColI^+ ^cells than either CD34^+^/ColI^+ ^or CD45^+^/ColI^+ ^cells and slightly more CD34^+^/ColI^+ ^cells than CD45^+^/ColI^+ ^cells (Figure 1D). The difference between CXCR4 and CD45 was statistically significant (*P *< 0.05), and the difference between CXCR4 and CD34 approached statistical significance (*P *= 0.07). The relative lack of CD45^+ ^fibrocytes is most likely due to the loss of CD45 that occurs once fibrocytes enter the lung [1,17].

### CXCR4 and CXCL12 are upregulated in the lungs of SSc-ILD patients

**Figure 2:**
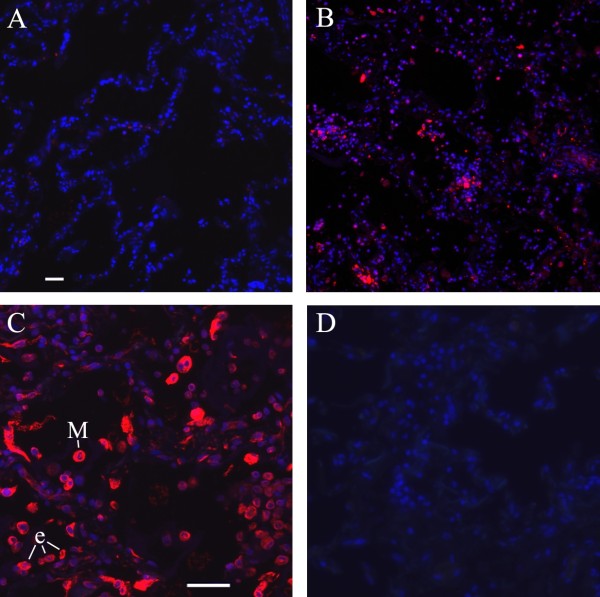
**Stromal cell-derived factor 1 in human lung tissue**. Tissue sections of normal and SSc-ILD lungs were stained with anti-stromal cell-derived factor 1 (anti-CXCL12) (red) and the nuclear stain DAPI (blue). **(A) **Normal lung tissue section at × 10 magnification. **(B) **SSc-ILD lung tissue section at × 10 magnification. **(C) **SSc-ILD lung tissue section at × 20 magnification. **(D) **SSc-ILD lung tissue section at × 20 magnification without primary antibody control. Note the massive CXCL12 staining in SSc-ILD lung tissue **(B) **and **(C) **and little staining in normal lung tissue **(A)**. e, epithelial cells; M, macrophage. Bars = 10 μm.

Because the most common fibrocyte subset in SSc-ILD lung tissue was positive for CXCR4, we compared the expression of CXCR4 in SSc and normal lung tissues. Western blot analysis showed a more than fourfold increase in CXCR4 levels (*P *< 0.001) in SSc-ILD tissue compared to normal lung tissue (Figures 1E and 1F). We also compared the expression of the CXCR4 ligand CXCL12 in SSc and normal lung tissue. Little if any staining was observed in control lung tissue sections (Figure 2A). CXCL12 was strikingly upregulated in SSc-ILD lung tissue (Figure 2B), both in cells with the morphological characteristics of hyperplastic epithelial cells lining the remaining airspaces and in alveolar macrophages (Figure 2C). No staining was detected in the absence of primary antibody (Figure 2D).

### Circulating fibrocyte subsets in SSc-ILD patients

**Figure 3:**
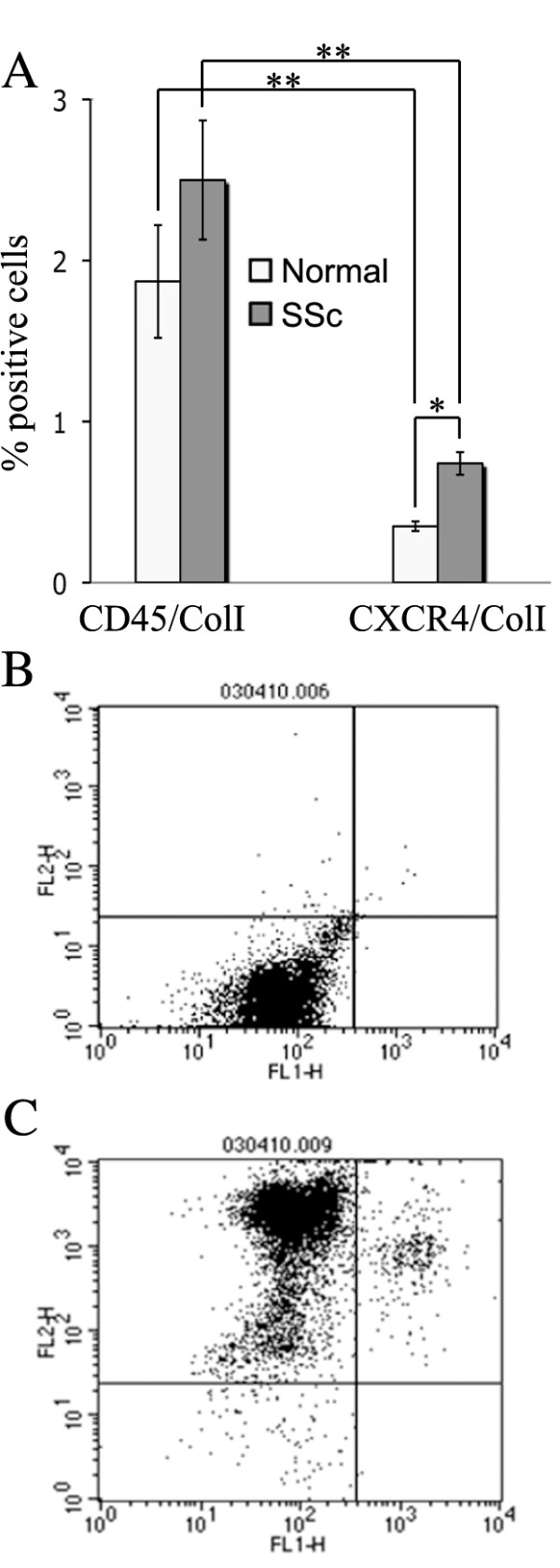
**Fibrocyte subsets in the peripheral blood of SSc-ILD patients and normal subjects**. Human peripheral blood mononuclear cells (PBMCs) were isolated and analyzed by flow cytometry for CD45, CXCR4 and ColI. Seven patients and seven normal subjects were used in these studies. **(A) **Note that the number of CD45^+^/ColI^+ ^and CXCR^+^/ColI^+ ^cells is higher in SSc-ILD blood than in normal blood. Note that CD45^+^/ColI^+ ^fibrocytes are much more prevalent than CXCR4^+^/ColI^+ ^cells. **P < 0.01. *P < 0.05. **(B) **and **(C) **Primary data demonstrating that fluorescence levels provided by isotype controls are readily distinguished from fluorescence levels provided by specific antibodies. For FL1 fluorescence, primary antibodies are biotinylated isotype controls **(B) **and biotinylated rabbit anti-ColI **(C)**. For FL2 fluorescence, antibodies are isotype control phycoerythrin (PE) **(B) **and anti-CD45 PE monoclonal antibody **(C)**.

Given the large number of fibrocytes in SSc lung tissue, we wanted to determine the prevalence of these cells in the peripheral blood of SSc patients. We found a greater number of CD45^+^/ColI^+ ^and CXCR4^+^/ColI^+ ^cells in the blood of SSc patients than in that of control subjects (Figure 3). Interestingly, while CXCR4^+^/ColI^+ ^cells were more prevalent than CD45^+^/ColI^+ ^fibrocytes in the lung tissue of SSc patients, the reverse was true in the peripheral blood. These results suggest that CXCR4^+^/ColI^+ ^cells may be relatively underrepresented in the blood of SSc patients because they migrate rapidly and efficiently into injured lung tissue.

### *SSc blood monocytes exhibit enhanced CXCL12-induced chemotaxis *in vitro

**Figure 4:**
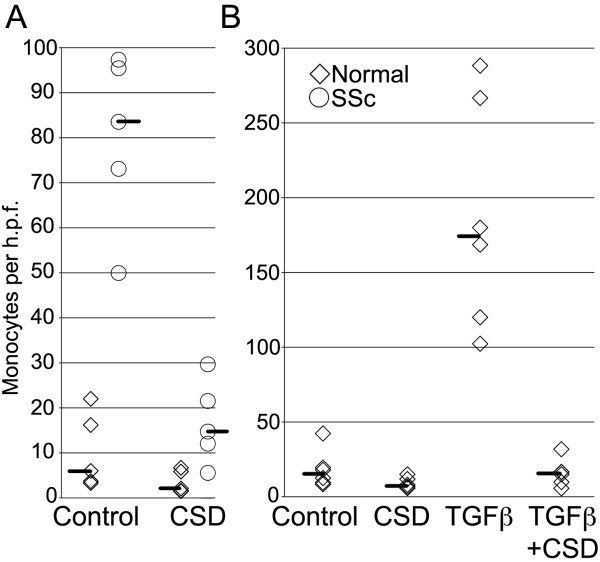
**Monocyte migration *in vitro***. The migration of normal and SSc-ILD monocytes toward CXCL12, the effect of TGFβ on normal monocyte migration and the ability of caveolin scaffolding domain (CSD) peptide to inhibit migration were quantified as described in Materials and methods. Each symbol represents the results obtained with cells from a different donor. The median value obtained in each category is indicated by a line. **(A) **Normal versus SSC-ILD ± CSD. Each symbol represents one subject. Note the almost sevenfold increase in migration in SSc-ILD lung tissue and the almost complete inhibition of migration by CSD peptide. **(B) **Normal ± transforming growth factor β (TGFβ) ± CSD. Each symbol represents one subject. Note the 15-fold increase in migration induced by TGFβ and the almost complete inhibition of migration by CSD peptide.

Since fibrocytes are derived from monocytes, SSc monocytes overexpress CXCR4 and CXCR4 mediates the migration of monocytes and fibrocytes into injured lung tissue [8], we used micro-Boyden chambers (in which cells placed in the upper chamber migrate through a membrane toward the chemoattractant placed in the lower chamber) to compare the migration of SSc and normal monocytes in response to CXCL12. The rate of migration toward CXCL12 was enhanced almost sevenfold in SSc monocytes compared to normal control monocytes (Figure 4 and Table 1) (*P *< 0.0001).

**Table 1:** CSD peptide inhibits monocyte migration toward CXCL12^a^

Source	TGFβ	CSD Peptide	Cells/high-power field (Mean ± SD)
Normal	-	-	12.0 ± 3.7
Normal	-	^+^	3.6 ± 1.1
Normal	^+^	-	187.7 ± 30.9
Normal	^+^	^+^	15.9 ± 3.6
SSc	-	-	80.0 ± 8.6
SSc	-	^+^	16.7 ± 4.1

^a^CSD, caveolin scaffolding domain; CXCL12, stromal cell-derived factor 1; TGFβ, transforming growth factor β; SSc, systemic sclerosis. These data summarize the results presented in Figure 4.

To confirm and expand on these findings, we next evaluated monocytes in which the effects of the underexpression of caveolin-1 (for example, overexpression of CXCR4) were reversed by treating cells with CSD peptide. CSD peptide almost completely inhibited the migration of both SSc and normal monocytes in response to CXCL12 (Figure 4 and Table 1) (*P *< 0.0001). In addition, we also examined the migration of normal monocytes in which the SSc phenotype (low caveolin-1, high CXCR4) was induced by treatment with TGFβ. TGFβ treatment enhanced the migration of normal monocytes 15-fold, and CSD peptide treatment almost totally reversed the enhancement of migration by TGFβ (Figure 4 and Table 1) (*P *< 0.0001). In summary, these data suggest that the recruitment of monocytes and fibrocytes into the lung tissue of SSc patients is mediated by the CXCR4/CXCL12 axis, which in turn is regulated by TGFβ and caveolin-1 [13,18].

### SSc peripheral blood cell phenotypes

**Figure 5:**
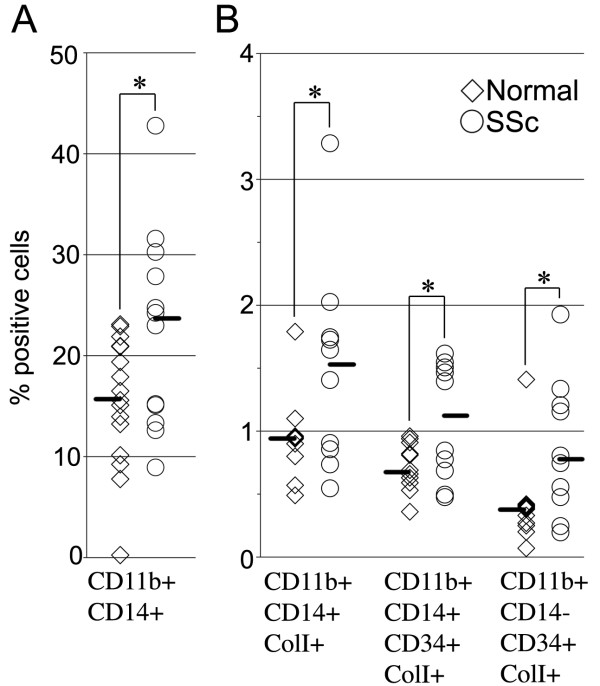
**Phenotype of SSc-ILD and normal monocytes**. PBMCs were isolated and analyzed by using flow cytometry as indicated for their expression of ColI, CD11b, CD14 and CD34. Each symbol represents the results obtained with cells from a different donor. The median value obtained in each category for SSc-ILD patients and normal donors is indicated by a line. **(A) **Double-labeled analysis. Note that among CD11b^+ ^monocytes, CD14^+ ^cells are more prevalent among SSc-ILD cells than among normal cells. **(B) **Triple- and quadruple-labeled analyses. Note that CD14^+^/ColI^+^, CD14^+^/CD34^+^/ColI^+^, and CD14^-^/CD34^+^/ColI^+ ^cells are more prevalent among SSc-ILD CD11b^+ ^monocytes than among normal CD11b^+ ^monocytes. *P < 0.05.

Given the altered migratory function of SSc monocytes, we evaluated whether their phenotype might also be altered. These studies revealed striking differences between normal and SSc CD11b^+ ^cells. A higher percentage of CD11b^+ ^SSc PBMCs CD11b^+ ^normal PBMCs was CD14^+ ^(Figure 5A) (*P *< 0.05). When we characterized these cells in terms of ColI and CD34 expression, we found statistically significant differences (*P *< 0.05) between scleroderma and normal PBMCs in CD11b^+^/CD14^+^/ColI^+^-expressing, CD11b^+^/CD14^+^/CD34^+^/ColI^+^-expressing and CD11b^+^/CD14^-^/CD34^+^/ColI^+^-expressing cells (Figure 5B). These observations indicate a major increase in the percentage of both fibrocytes and collagen-producing monocytes among SSc-ILD patients compared to normal control subjects. The data are particularly striking for classic fibrocytes (CD11b^+^/CD14^-^/CD34^+^/ColI^+ ^cells), in which eight of the nine subjects with the highest levels of these cells were SSc-ILD patients. Our data are in agreement with the recent observation that CD45^+^/CD34^+^/CD14^+^/Col I^+ ^cells are present in the peripheral blood of SSc patients [7]. We also evaluated the possibility that ColI^+ ^cells could be CD14^-^/CD34^-^. Indeed, there are CD11b^+^/ColI^+^/CD14^-^/CD34^- ^circulating cells, and the levels of these cells are higher in SSc patients than in controls, but the difference does not quite reach statistical significance (data not shown).

### CD14^+^/ColI^+ ^cells are present in the lung tissue of SSc patients but not of healthy individuals

**Figure 6:**
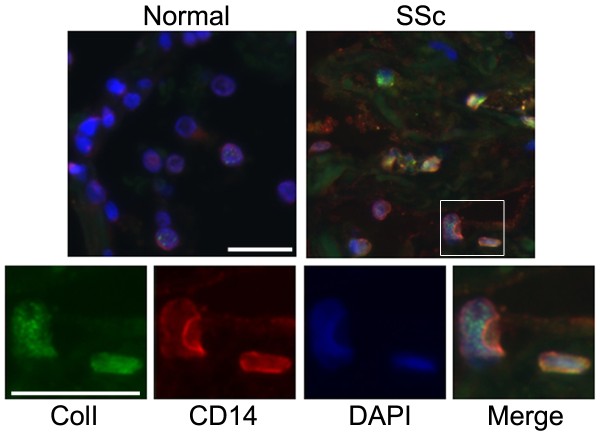
**CD14^+^/ColI^+ ^cells are present in SSc-ILD lung tissue**. Fixed, paraffin-embedded adult lung tissue sections from normal and SSc-ILD subjects were stained for CD14 and ColI. Nuclei were stained with DAPI (blue). Top: low-magnification images showing SSc-ILD adult lung tissue. Bottom: high-magnification images showing SSc-ILD adult lung tissue. The portion of the low-magnification image presented at high magnification is boxed. ColI, green; CD14, red. Note that CD14^+^/ColI^+ ^cells are present in SSc-ILD lung tissue, but not in normal lung tissue. Bar = 10 μm.

To determine whether CD14^+^/Col I ^+ ^cells (see above) exist in SSc patients *in vivo*, we examined lung tissue for the presence of these cells. We identified CD14^+^/ColI^+ ^cells in all seven of seven lung tissue samples obtained from SSc-ILD patients, while no cells with this phenotype were found in the lung tissue samples from healthy individuals (Figure 6). Specifically, 20.9 ± 5.8 CD14^+^/ColI^+ ^cells were observed per × 20 microscopic field (seven patients with six randomly chosen fields per slide) compared to none of four normal lung tissue samples. These data further support our *in vitro *findings and confirm the abnormality of the monocyte-fibrocyte-fibroblast lineage in SSc.

### CSD peptide treatment inhibits fibrocyte accumulation in the lungs of bleomycin-treated mice

**Figure 7:**
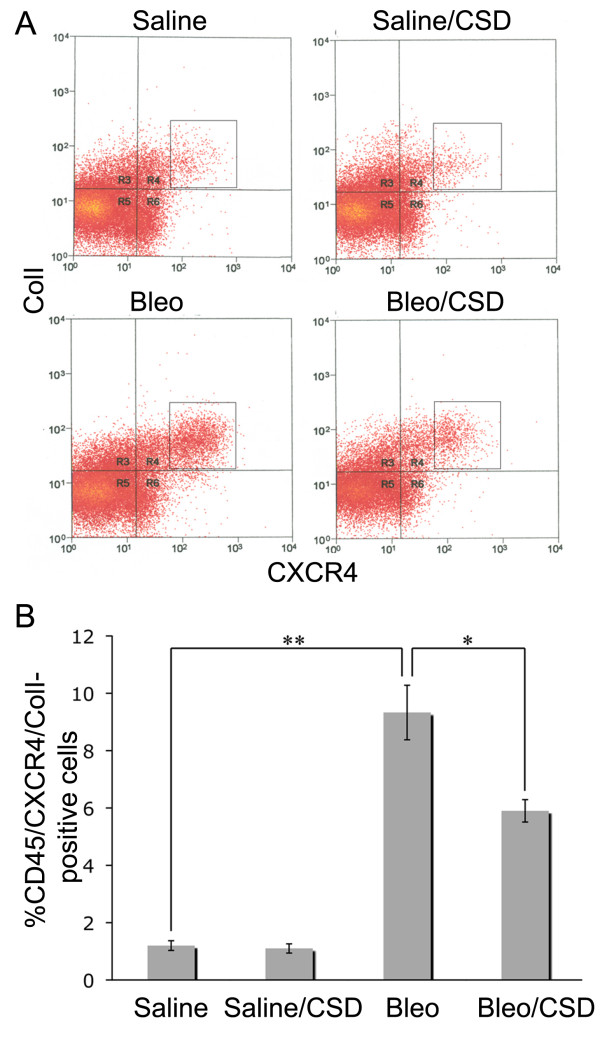
**Flow cytometric quantification of CD45^+^/CXCR4^+^/ColI^+ ^fibrocytes in mouse lung tissue**. Total lung cells were isolated from mice (three to six mice in each category) ten days after saline or bleomycin treatment. The mice had been treated daily with CSD or control peptide starting one day prior to saline or bleomycin treatment. CD45^+^/CXCR4high/ColI^+ ^fibrocytes were quantified by performing flow cytometry. **(A) **Results from a typical experiment in which lung cells from the indicated individual mice were gated based on CD45 staining, then resolved on two-dimensional scatterplots based on their ColI and CXCR4 staining. Vertical and horizontal bars delimit the zone in which cells stained with isotype controls are found. We refer to the cells that are slightly into the upper right quadrant as CXCR4low, and we refer to cells within the box as CXCR4high. Therefore, the square box contains cells that are CD45^+^/CXCR4high/ColI^+^. **(B) **The percentage of cells falling within the square box is presented for each category of mice. The data represent the average of from three to six mice in each category ± standard error of the mean. **P < 0.01. *P < 0.05.

**Figure 8:**
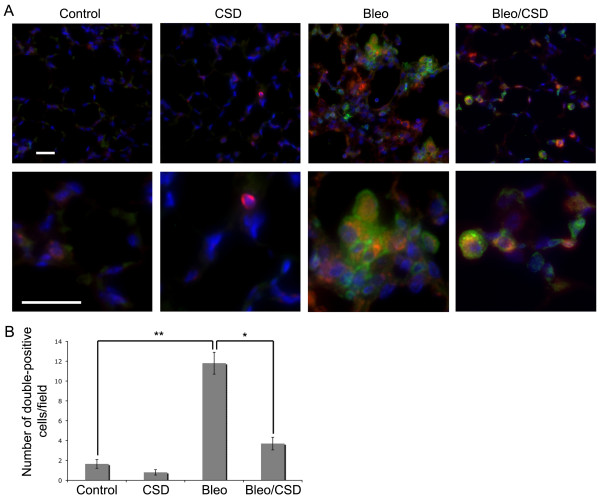
**CSD peptide inhibits the bleomycin-induced accumulation of CD45^+^/ColI^+ ^fibrocytes in lung tissue**. Sections from saline-treated mice receiving the CSD peptide (CSD) or the control, scrambled peptide (Control) and sections from bleomycin-treated mice receiving the CSD peptide (Bleo/CSD) or the scrambled peptide (Bleo) were harvested ten days after treatment, then stained for CD45 (green) and ColI (red). Nuclei were labeled with DAPI (blue). **(A) **Upper row: merged low-magnification images. Lower row: merged high-magnification images. Bars = 10 μm. **(B) **The average number of CD45^+^/ColI^+ ^fibrocytes per field was determined in images from six mice in each category, with five fields per mouse. **P < 0.01. *P < 0.05.

We have shown that CXCR4^+ ^fibrocytes accumulate in the lung tissue of SSc patients (Figure 1) and that CSD peptide treatment inhibits the overexpression of CXCR4 by SSc monocytes [13] and their enhanced migration toward CXCL12 *in vitro *(Figure 4). To determine whether the same mechanisms operate *in vivo*, we quantified fibrocytes in the lung tissue of bleomycin-treated mice and the effect of CSD peptide treatment. While there is a population of cells that are slightly CXCR4^+ ^(CXCR4low) that are present at similar levels in both saline-treated and bleomycin-treated mice, we consider fibrocytes to be the population of CD45^+^/ColI^+^/CXCR4high cells in the boxes that are present at much higher levels in bleomycin-treated mice than in saline-treated mice. Flow cytometric studies showed that the percentage of CD45^+^/CXCR4high/Col I^+ ^cells was increased more than fivefold by bleomycin treatment and that this increase was reduced by about 50% by CSD peptide treatment (Figure 7). While few CD45^+^/Col I^+ ^cells were observed in tissue sections from saline-treated mice, bleomycin induced a fivefold increase in the number of CD45^+^/Col I^+ ^cells (Figure 8). CSD peptide treatment significantly inhibited this increase by about 75%.

## Discussion

Fibrocyte accumulation in lung tissue or peripheral blood has been observed in a variety of fibrotic lung diseases, including IPF [12,18-20], SSc [7,21], asthma [22] and COPD [23]. In the current study, we have confirmed and expanded upon recent observations regarding SSc-ILD fibrocytes [7]. Most important, we have determined that the accumulation of fibrocytes in SSc-ILD lung tissue may relate to the altered phenotype and migratory activity of their monocyte precursors.

We observed fibrocytes in the lung tissue of SSc-ILD patients, but not that of healthy individuals. We used three different hematopoietic markers in these experiments (in addition to the mesenchymal marker ColI) and found the order of prevalence among fibrocyte subsets to be CXCR4^+^/ColI^+ ^> CD34^+^/ColI^+ ^> CD45^+^/ColI^+^. Our data regarding fibrocyte accumulation and fibrocyte subsets in lung tissue are similar to observations made regarding samples from IPF patients [12]. The fibrocyte levels in peripheral blood are also similar in IPF and SSc-ILD (about threefold enhancement in IPF [24] and about twofold enhancement in SSc-ILD patients), although a 15-fold enhancement was observed in IPF during acute exacerbations. Unlike our observations in the lung, we detected fewer CXCR4^+^/ColI^+ ^cells than CD45^+^/ColI^+ ^cells in the peripheral blood of SSc-ILD patients. These observations are consistent with the notion that the high level of CXCL12 we observed in the lung tissue of SSc-ILD patients, combined with the high level of CXCR4 we observed [13] in SSc-ILD monocytes (that is, fibrocyte precursors), leads to a relatively low steady-state number of CXCR4^+^/ColI^+ ^cells in the peripheral blood and their accumulation in the lungs. A similar situation is also observed in bleomycin-treated mice, in which there are very high levels of fibrocytes in the lungs and low levels in the circulation [8]. In summary, analyses of samples from IPF patients [9,19], SSc-ILD patients [13] and mice treated with bleomycin [8,18] (considered to be a model for both IPF and SSc-ILD) demonstrate the central role of the CXCL12/CXCR4 axis in the recruitment of fibrocytes into damaged lung tissue.

We performed migration assays using CXCL12 as a chemoattractant to directly examine the role of the CXCL12/CXCR4 axis in regulating the migration of monocytes in SSc-ILD. We previously showed that SSc-ILD monocytes overexpress CXCR4 because of a deficiency in the master signaling molecule caveolin-1 and that this phenotype (that is, low caveolin-1 and high CXCR4) can be generated in normal monocytes by treatment with TGFβ. In accord with their overexpression of CXCR4, we report herein that both SSc-ILD monocytes and TGFβ-treated normal monocytes demonstrate extremely high degrees of migration in response to CXCL12. In both cases, CSD peptide treatment, which compensates for the lack of caveolin-1 and thereby inhibits CXCR4 expression, almost completely inhibited migration.

While we would like to have performed similar migration experiments using normal and SSc-ILD fibrocytes produced by differentiation of monocytes *in vitro*, our current observations identified differences between the starting normal and SSc PBMC populations that made these experiments too complex to perform and interpret as part of the current study. These complexities are related to ambiguities in the definition of fibrocytes. Fibrocytes are defined as cells that express both hematopoietic markers and fibroblast markers. While all investigators agree on CD45 and CXCR4 as hematopoietic cell markers and ColI as a fibroblast marker, the use of CD14 and CD34 as hematopoietic cell markers is controversial.

The data dealing with CD14 are complex. It has been proposed that CD14^+ ^monocytes (but not CD14^- ^monocytes) under the influence of factors secreted by T cells and B cells differentiate into spindle-shaped, CD14^-^, ColI^+ ^fibrocytes. Other investigators dispute the idea that the precursors must be CD14^+ ^[18,25]. Results from our laboratory and others [7,26] indicate that CD45^+^/ColI^+^/CD14^+ ^cells exist and are particularly prevalent in samples from SSc-ILD patients. We find that the subpopulations of SSc-ILD PBMCs that are CD11b^+^/CD14^+ ^or CD11b^+^/CD14^+^/ColI^+ ^are much greater than those found among normal PBMCs. The greater fraction of CD14^+ ^cells raises the possibility that the percentage of SSc-ILD monocytes able to differentiate into fibrocytes is higher than the percentage for normal monocytes. The greater fraction of CD14^+^/ColI^+ ^cells suggests that SSc-ILD monocyte preparations may contain a significant number of collagen-producing monocytes or cells that are partially differentiated into fibrocytes. The physiological relevance of CD14^+^/ColI^+ ^cells in the pathogenesis of SSc-ILD is further supported by our observation that these cells are present in the lung tissue of SSc-ILD patients, but not that of control subjects.

In contrast to CD14, CD34 is not present on normal monocytes [27]. Its presence on fibrocytes is controversial, with some investigators reporting that it is present [20] and others reporting that it is absent [28] on normal fibrocytes. Our observations regarding CD34 are (1) that CD34^+^/ColI^+ ^cells are prevalent in the lung tissue and peripheral blood of SSc-ILD patients, and (2) in agreement with other investigators [7,26], these CD34^+ ^peripheral blood cells from SSc-ILD patients can be either CD14^+ ^or CD14^-^. In summary, returning to cell migration, to perform a complete analysis comparing the migratory ability of normal and SSc fibrocytes, it would be necessary to (1) separate CD14^+ ^and CD14^- ^monocytes from each source, and (2) after the differentiation of these populations *in vitro *into spindle-shaped cells, separate these populations into CD14^+ ^and CD14^- ^cells. In this manner, we could distinguish whether differences in the ability of SSc-ILD and normal fibrocytes and collagen-producing monocytes to migrate is due to their source (for example, SSc-ILD versus normal) or to their phenotype (for example, CD14^+ ^versus CD14^-^).

We previously showed a variety of beneficial effects of CSD peptide treatment on bleomycin-induced lung fibrosis, including improved histopathology, inhibition of alveolar epithelial apoptosis and inhibition of monocyte accumulation in the lungs. We also previously showed that SSc-ILD monocytes overexpress the promigratory cytokine receptor CXCR4 on the basis of their underexpression of caveolin-1 and that their overexpression of CXCR4 is reversed by CSD peptide treatment. Given that CXCR4 is the primary cytokine receptor on fibrocytes, that CXCR4 mediates the migration of fibrocytes into the lung tissue of bleomycin-treated mice [8] and that inhibiting the expression of the CXCR4 ligand CXCL12 inhibits fibrocyte migration into the lung tissue of bleomycin-treated mice [8], we used the bleomycin model to examine the effects of CSD peptide on fibrocyte migration *in vivo*. In agreement with the results of our *in vitro *studies, *in vivo *CSD peptide treatment inhibited the accumulation of fibrocytes in the lung tissue of these mice. The combined observations suggest that CSD peptide treatment provides protection against bleomycin-induced lung fibrosis in large part by inhibiting the expression of CXCR4 by monocytes and fibrocytes and thereby inhibiting their migration into damaged lung tissue.

## Conclusion

Our data strongly support the idea that fibrocytes and collagen-producing monocytes play a critical role in the pathology of SSc-ILD and that these cells may differentiate aberrantly and accumulate in the lungs because of the altered phenotype and migratory properties of SSc-ILD monocytes and monocyte-derived cells. Whether fibrocytes and collagen-producing monocytes are distinct cell types or whether they are simply variations on the fibrocyte theme (that is, cells expressing a hematopoietic marker and ColI) is an open question and possibly just one of semantics. In other words, a fibrocyte by any other name would "smell as sweet." These studies further highlight the potential ability of CSD peptide to serve as a treatment for fibrotic lung diseases by inhibiting the differentiation of fibrocytes and their migration into damaged lung tissue.

## Materials and methods

### Patients

Under a protocol approved by the Institutional Review Board for Human Research for a Rheumatology Research Repository, patients with SSc-ILD were recruited from the Scleroderma Clinic at the Medical University of South Carolina (MUSC). All patients fulfilled the American College of Rheumatology (formerly the American Rheumatism Association (ARA)) criteria for SSc [29]. Nine were classified as having diffuse cutaneous SSc, and four were classified as having SSc-overlap according to previously defined standards [13]. All 13 patients had evidence of SSc-ILD as previously defined [13]. Demographic data are summarized in Table 2.

**Table 2:** Clinical features of SSc patients^a^

Patient characteristics	Patients	Controls
Caucasian males, *n*	3	9
Caucasian females, *n*	4	12
African-American males, *n*	1	2
African-American females, *n*	4	2
Asian females, *n*	1	1
Smokers, *n*	1	0
Former smokers, *n*	3	0
Mean age ± SD, years (range)	54.6 ± 9.8 (39 to 75)	43.7 ± 9.4 (22 to 57)
Disease: limited cutaneous, *n*	0	Not applicable
Diffuse cutaneous	9	Not applicable
Overlap	4	Not applicable
Disease duration, years: mean ± SD (range)	9.4 ± 6.6 (1 to 22)	Not applicable
Pulmonary involvement (ILD)	13 of 13 (100%)	Not applicable
Pulmonary Hypertension	5 of 13 (38.5%)	Not applicable
GI involvement	12 of 13 (92.3%)	Not applicable
Cardiac involvement	7 of 13 (53.8%)	Not applicable
Renal involvement	0 of 13 (0%)	Not applicable
Autoantibodies: ANA^+^	11 of 11 (100%)	Not applicable
Scl-70^+^	4 of 11 (36%)	Not applicable
Anticentromere	0 of 10 (0%)	Not applicable

^a^SSc, systemic sclerosis; SD, standard deviation; ILD, interstitial lung disease; GI, gastrointestinal; ANA, antinuclear antibody.

Normal human lung tissue was obtained from the Brain and Tissue Bank for Developmental Disorders (Baltimore, MD, USA) or from the National Disease Research Interchange (Philadelphia, PA, USA). Under a protocol approved by the MUSC Institutional Review Board for Human Research, SSc lung tissue was obtained from autopsy specimens collected by the MUSC Division of Pathology and Laboratory Medicine. Tissue was obtained from six female SSc patients (five white and one African-American) and one African-American male SSc patient, all of whom had evidence of SSc-ILD (Table 3).

**Table 3:** Clinical features of autopsy samples^a^

Patient characteristics	Patients	Controls
Caucasian males, *n*	0	2
Caucasian females, *n*	5	2
African-American males, *n*	1	0
African-American females, *n*	1	0
		
Smokers, *n*	1	0
Former smokers, *n*	1	0
		
Mean age ± SD (range)	53.6 ± 11.4 (38 to 65)	51.3 ± 22.4 (23 to 77)
Disease: limited cutaneous, *n*	5	Not applicable
Diffuse cutaneous, *n*	2	Not applicable
Overlap, *n*	0	Not applicable
Pulmonary involvement (ILD), *n *(%)	7 of 7 (100%)	Not applicable
GI involvement, *n *(%)	5 of 7 (71.4%)	Not applicable
Cardiac involvement, *n *(%)	5 of 7 (71.4%)	Not applicable
Renal involvement, *n *(%)	1 of 7 (14.3%)	Not applicable

^a^SD, standard deviation; ILD, interstitial lung disease; GI, gastrointestinal.

### Immunohistochemistry

Immunohistochemistry of human and mouse lung tissue sections was performed as previously described [14]. Briefly, paraffin-embedded sections were stained with primary antibodies, appropriate Alexa Fluor 488- or Alexa Fluor 555-conjugated secondary antibodies (Invitrogen, Carlsbad, CA, USA) and the nuclear stain 4', 6-diamidino-2-phenylindole (DAPI) (Invitrogen). Images were collected using a Leica DMI 4000 B fluorescence microscope (Leica Microsystems, Inc., Buffalo Grove, IL, USA). Primary antibodies were rat anti-human (and mouse) CD45 and rat anti-human CXCR4 (BD Pharmingen, San Jose, CA, USA, rabbit anti-human (and mouse) CXCR4 (Santa Cruz Biotechnology, Santa Cruz, CA, USA), rabbit anti-mouse ColI (Cedarlane USA, Burlington, NC, USA), rat anti-human (and mouse) CD14 (Biolegend, San Diego, CA, USA), mouse anti-human CXCL12 (R&D Systems, Minneapolis, MN, USA) and a rabbit antibody against the human ColI type α1 C-terminal propeptide prepared in our laboratory.

### Western blot analysis

Western blot analyses of human lung tissue were performed as previously described [13,14].

### PBMC isolation and monocyte enrichment

Human PBMC isolation and enrichment of monocytes was performed as previously described [13]. Briefly, 40 mL of peripheral blood was drawn from SSc patients and healthy individuals. Blood treated with anticoagulant was diluted 1:2 with Hank's Balanced Salt Solution and centrifuged on a 1.083 g/mL Histopaque cushion (Sigma, St Louis, MO, USA). PBMCs were harvested from the interface by centrifugation. Monocytes were enriched from total PBMCs by immunodepletion using a Dynal Monocyte Negative Isolation Kit (Invitrogen).

### Peptide treatments

Treatment with CSD peptide and control peptides was performed as previously described [14]. The CSD peptide (amino acids 82 to 101 of caveolin-1; DGIWKASFTTFTVTKYWFYR) was synthesized as a fusion peptide to the C terminus of the Antennapedia Internalization Sequence (RQIKIWFQNRRMKWKK). Either scrambled CSD attached to the Antennapedia Internalization Sequence or the Antennapedia Internalization Sequence alone was used as a control peptide. Neither control peptide ever showed any effect.

### Cell migration

Migration of normal and SSc monocytes was assessed as described previously [30] with modifications. Briefly, chemoattractant CXCL12 at 100 ng/mL in RPMI 1640 medium with 1% BSA (Sigma) was placed into the lower wells of Neuro Probe Multiwell Chemotaxis Chambers (Neuro Probe, Gaithersburg, MD, USA). A quantity of 25 μL of cell suspension (1 × 10^6 ^monocytes/mL) with or without 10 ng/mL TGFβ pretreatment (45 minutes in RPMI 1640 medium with 1% BSA) was placed in the upper wells. CSD or control peptide (5 μmol final concentration) was added to cells before they were placed in the upper chamber. A 5-μm pore size polycarbonate filter was used to separate the two compartments. After incubation for 3 hours at 37°C in humidified air with 5% CO_2_, the filter was removed, fixed and stained with DAPI (Invitrogen). The cells on the underside of the membrane were photographed and counted in six high-power fields per filter.

### Flow cytometry

Human PBMCs were analyzed as described previously [9,10] with modifications. Briefly, to label cell-surface markers, cells (1 × 10^5 ^per aliquot) in PBS containing 01% BSA and 0.1% sodium azide (fluorescence-activated cell sorter (FACS) buffer) were incubated with one or more of phycoerythrin (PE)-CD11b, CD14 PerCP, CD34 allophycocyanin (APC) (all from BD Pharmingen), CD45 PE (BD Biosciences, San Jose, CA, USA) and CXCR4 APC (R&D Systems), or with labeled isotype control antibodies for 30 minutes at 4°C. Next, cells were permeabilized using a cytofix/cytoperm kit (BD Biosciences). ColI was labeled using biotinylated anti-ColI (Rockland Immunochemicals Inc., Gilbertsville, PA, USA) and fluorescein isothiocyanate-conjugated streptavidin (BD Pharmingen). Biotinylated isotype control antibody was also obtained from Rockland Immunochemicals Inc. After the cells were washed in FACS buffer, fluorescence data were acquired on a FACSCalibur flow cytometer and analyzed using BD CellQuest software (both from BD Biosciences). At least 10,000 cells were analyzed per condition.

### Bleomycin experiments

Bleomycin-induced lung injury and CSD peptide treatment were performed as previously described [13]. This procedure was approved by the MUSC Institutional Animal Care and Use Committee. Ten days after bleomycin treatment one group of mice was killed for immunohistochemical analysis of lung tissue sections as described above. Images were acquired using a Zeiss 510SML Laser Confocal Microscope (Carl Zeiss, Thornwood, NY, USA).

Ten days after treatment with bleomycin or saline vehicle lungs were removed, diced and digested with collagenase for flow cytometry of fibrocytes in lung tissue [31]. Total lung cells were analyzed by flow cytometry as described above using antibodies against CD45 and CXCR4 prior to permeabilization and antibodies against ColI after permeabilization.

### Statistical analysis

Immunoreactive bands were quantified by densitometry using ImageJ version 1.32 software (National Institutes of Health, Bethesda, MD, USA). Raw densitometric data were processed and analyzed using GraphPad Prism 3.0 software (GraphPad Software Inc., La Jolla, CA, USA). Protein expression was evaluated by performing Western blot analysis, immunohistochemistry or flow cytometry, and data were assessed using Student's *t*-test. In all figures, statistical significance is expressed as ***P < 0.001, **P < 0.01 and *P < 0.05.

## Abbreviations

BSA: bovine serum albumin; ColI: collagen I; CSD: caveolin-1 scaffolding domain; CXCR4: C-X-C chemokine receptor type 4; FACS: fluorescence-activated cell sorting; ILD: interstitial lung disease; PBS: phosphate-buffered saline; SDF-1: stromal cell-derived factor 1: also known as CXCL12; SEM: standard error of the mean; SSc: systemic sclerosis (scleroderma); TGFβ: transforming growth factor β.

## Competing interests

While none of the authors have so far received any financial benefit from the following, the Medical University of South Carolina has submitted a use patent on the caveolin-1 scaffolding domain peptide as a treatment for fibrotic diseases, and SH and ET are the founders of a company which expects to license this technology from MUSC and develop this drug.

## Authors' contributions

ET participated in study design, human and animal studies, data interpretation and manuscript preparation. MB participated in animal studies and manuscript preparation. JO participated in data interpretation and manuscript preparation. AH performed flow cytometry on human cells. MR and SZ performed immunohistochemical analyses on human tissues. RPV participated in study design, data interpretation and manuscript preparation. JZ performed animal studies. CMH analyzed patient demographics. RMS participated in data interpretation and manuscript preparation. SH participated in study design, data interpretation and manuscript preparation and performed statistical analyses. All authors read and approved the final manuscript.
